# Case report: Successful treatment of mediastinal unicentric castleman disease using cardiopulmonary bypass

**DOI:** 10.3389/fcvm.2023.1130237

**Published:** 2023-05-10

**Authors:** Wei Ran, Zhu Cuilin, Piao Hulin, Liu Kexiang

**Affiliations:** Dept Cardiovasc Surg, Second Hosp Jilin Univ, Changchun, China

**Keywords:** castleman's disease, middle mediastinal tumor, mixed pathology, cardiopulmonary bypass, surgery

## Abstract

Unicentric Castleman disease (UCD) is a rare, benign lymphoproliferative disorder. Mediastinal UCD has tumors with no clear boundaries that are highly vascularized. Resection surgery results in bleeding, leading to further challenges. Mixed-type UCD is rare. We report the case of a 38-year-old asymptomatic patient with mixed-type UCD; the tumor measured 7.8 cm in size and had unclear boundaries. The tumor was successfully resected by performing a cardiopulmonary bypass on the beating heart; the patient recovered uneventfully.

## Introduction

Castleman disease (CD), first reported by Castleman and Towne in 1954, is a rare, benign lymphoproliferative disorder (1). CD can be divided into two categories: unicentric Castleman disease (UCD) and multicentric Castleman disease (MCD). Mediastinal UCD is a common type of UCD that is accompanied by prominent feeding vessels. Owing to the lack of typical clinical signs, CD is difficult to diagnose. Herein, we report the case of a 38-year-old asymptomatic patient with mixed-type UCD; the tumor measured 7.8 cm in size and had unclear boundaries. The tumor was successfully resected by performing a cardiopulmonary bypass (CPB) on the beating heart.

## Case presentation

**Figure 1:**
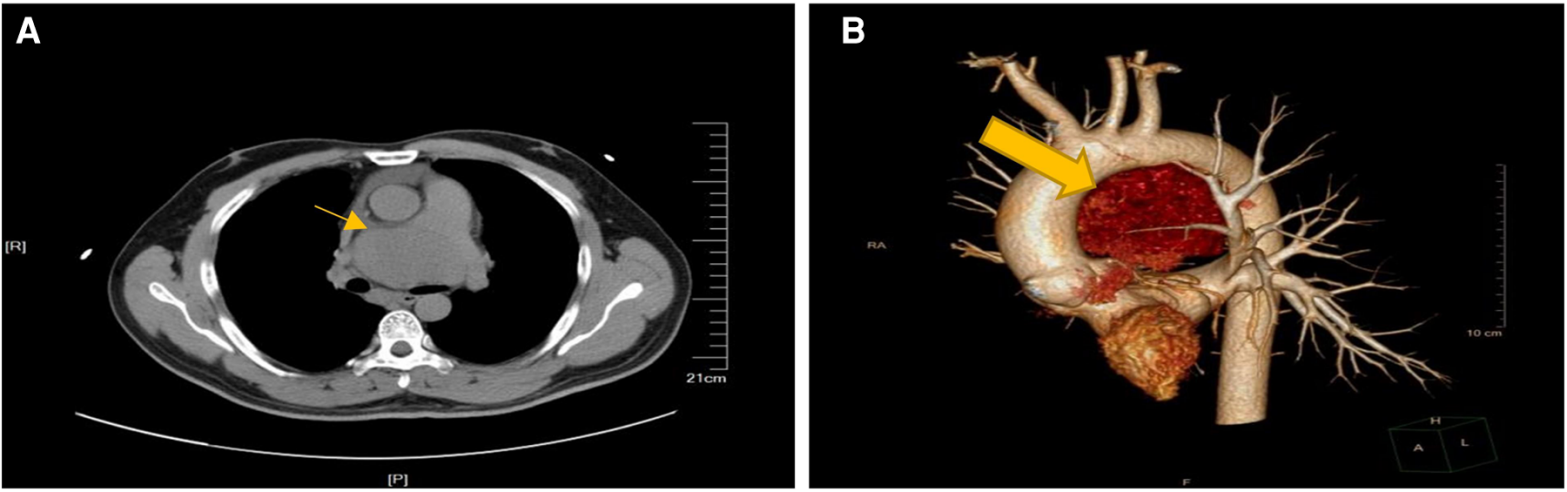
Preparative computed tomography scan of the chest showing in the anterior mediastinum. (**A**) The mass close to the main pulmonary artery bifurcation and extended along the right pulmonary artery. (**B**) Lateral view of 3-D computerized tomography showing the mass between aortic arch and pulmonary artery.

A 38-year-old man with no obvious symptoms was found to have a mediastinal tumor during a physical examination. The patient had no significant medical history except having diabetes mellitus for a year. He was referred to our hospital for medical treatment. No pathologic signs were found through other physical examinations. His laboratory test results were all within the normal limits, except for the lymphocyte count (13.1%), which was lower than the normal range. The levels of tumor markers alpha-fetoprotein, human chorionic gonadotropin, and lactate dehydrogenase were all normal. The thoracic computed tomography (CT) scan revealed a huge soft tissue density shadow of 72 × 48 mm in the mediastinum. The tumor was compressed near the main pulmonary artery bifurcation and extended along the right pulmonary artery and did not have clear boundaries (Figure 1). His cardiac ultrasonography revealed a huge pericardial effusion, approximately 28 mm. Considering the location of the tumor and the risk of bleeding, we did not schedule a biopsy for him. The tumor was planned for resection via median sternotomy.

After sternotomy, the tumor was observed to occupy the area within the aorta-pulmonary window, which also severely compressed the cardiac left atrium. The tumor was vascularized and thus was highly susceptible to bleeding. Disassociation of the pulmonary arteries and the left atrium was essential for successful and complete resection of the tumor. The tumor was huge, and dissection of the tumor was attempted from the pulmonary artery and the left atrium, but the patient was hemodynamically unstable. To guarantee the safety of the patient, the surgical team decided to complete the surgery by performing CPB on the beating heart as an adjunct surgical support. CPB was performed via cannulation of the femoral artery and superior and inferior vena cava. The pressure in the pulmonary artery and heart was reduced through CPB. The aorta and pulmonary artery were pulled away to expose the tumor completely, and then, the tumor was meticulously dissected along the margins from the surrounding tissues. Before dissociation, the area of strong adhesion was gently peeled off and the borders of the blood vessels and the surrounding tissue were carefully confirmed. Electrocoagulation was used to control bleeding from tiny bleeding spots, whereas ligation was done when bigger blood vessel branches were found. The tumor was completely resected after a CPB duration of 165 min. After recovery of stable hemodynamics, CPB was discontinued. The remainder of the surgery was smooth, including hemostasis and chest closure.

**Figure 2:**
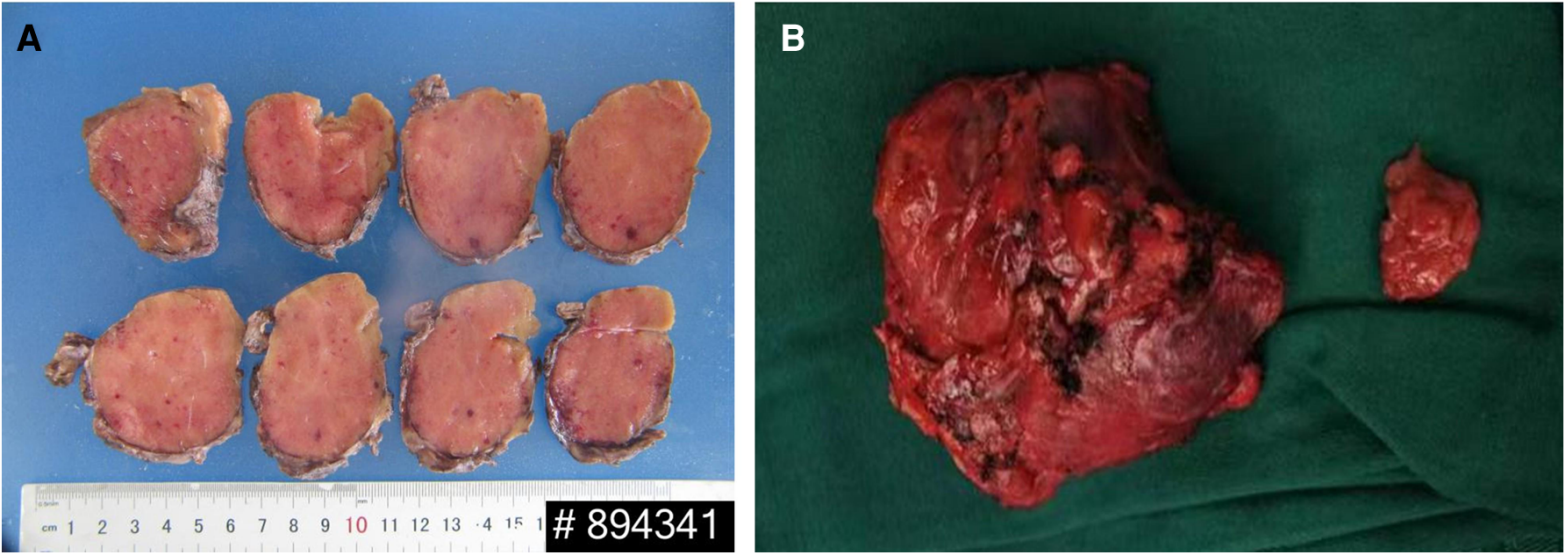
(**A**) The cut surface showing a solid, homogeneous, and gray mass. (**B**) Gross appearance of the mass.

**Figure 3:**
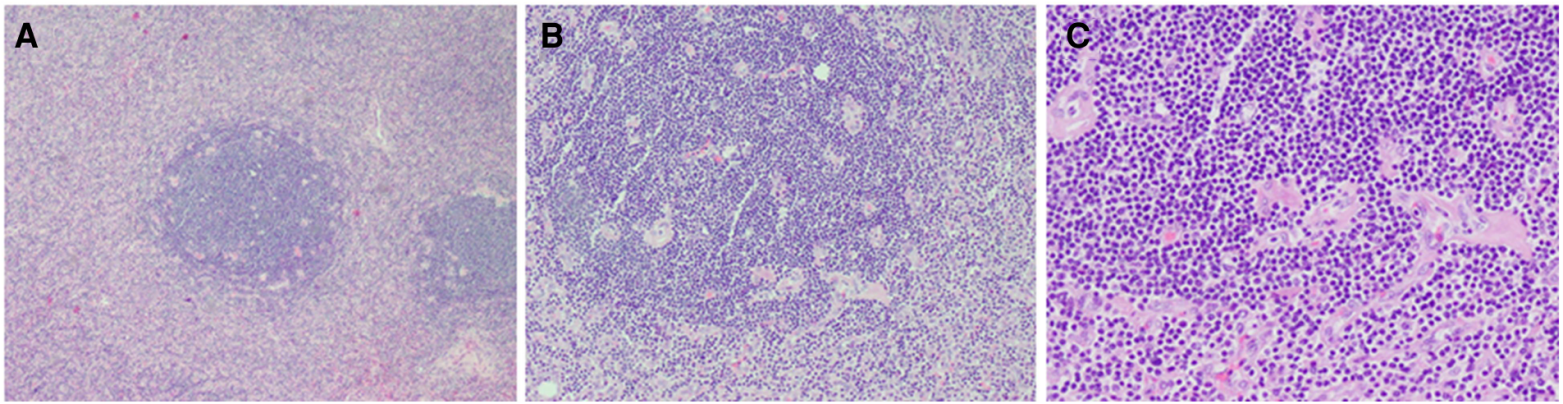
Histopathological examination showed (**A**) preserved lymph node architecture with a capsule, (**B**) surrounded by peripheral lymphocytes in concentric circles, (**C**) interfollicular vascular proliferation with perivascular hyalinization and plasma cells and histiocytes infiltration.

On gross inspection, the resected tumor measured 7.8 cm in size (Figure 2). Microscopic examination demonstrated preserved lymph node architecture with a capsule, surrounded by peripheral lymphocytes in concentric circles, interfollicular vascular proliferation with perivascular hyalinization, plasma cells, and macrophage infiltration (Figure 3). Immunohistochemical staining showed the following: CD20 (+), CD3 (+), PAX5 (+), CD5 (+), CyclinD1 (−), CD10 (−), Bcl6 (−), CD21 (FDC +), Kappa (+), Lambda (+), IgD (weak +), Ki67 (positive rate 15%), CD23 (FDC +), CD38 (+), CD30 (scattered +), MUMI (partial +), CD34 (vascular +), HHVV8 (−), and CD123 (−). Based on the pathology slides, the final diagnosis of this patient was mixed-type UCD.

**Figure 4:**
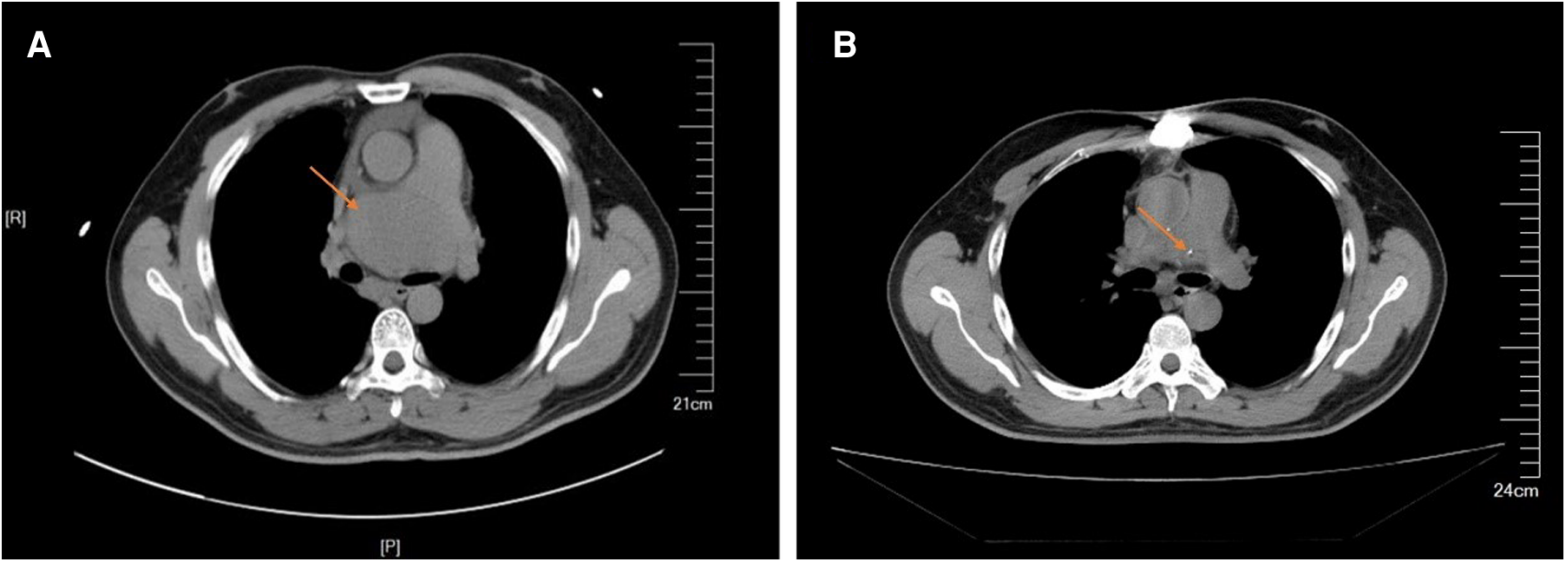
(**A**) Preparative computed tomography scan showed that the mass close to the main pulmonary artery bifurcation and extended along the right pulmonary artery. (**B**) Postoperative computed tomography scan showed no residual and no recurrence of the tumor.

The patient had an uneventful postoperative course and was discharged on postoperative day 21. The patient was continuously followed up; he was doing well 1 year postoperatively. The 1-year CT scan showed no residual tumor and no recurrence of the tumor (Figure 4). Regular annual CT imaging follow-up was advised for the patient.

## Discussion

CD is a rare, benign lymphoproliferative disorder of unknown etiology and is also called follicular lymph node hyperplasia. Pathologically, it can be divided into hyaline vascular type, plasma cell type, or mixed type. Clinically, single lymph node region enlargement is defined as UCD and multiple lymph node region enlargement as MCD. UCD is mostly hyaline vascular type, MCD is mostly plasma cell type and mixed type, and mixed-type UCD is rare (2). Our patient had mixed-type UCD, which is rare.

UCD is mostly asymptomatic, and chest tightness, dyspnea, cough, and dysphagia can occur when the tumor increases in size and starts oppressing the surrounding tissues and organs. UCD often occurs in the mediastinum and needs to be differentiated from thymoma, lymphoma, metastatic tumor, and neurogenic tumor. Pericardial effusion is associated with CD (3, 4), possibly due to the generalized inflammatory syndrome related to plasma cell histology. The pathological type of our patient was categorized as mixed type, and pericardial effusion is not a common clinical manifestation of mixed type. To the best of our knowledge, this is the first case of mixed-type UCD presenting with pericardial effusion as a clinical manifestation.

Surgical excision of the tumor is both a diagnostic and therapeutic procedure. The gold standard of diagnosis is pathological examination (5). Although ultrasound and CT can be used as diagnostic tools, ultrasound-guided penetration has the risk of causing hemorrhage because UCD is densely packed with blood vessels (6). Our team believes that preoperative puncture has a high risk of bleeding; thus, biopsy during surgery was considered to be safer. Therefore, in our case, we did not perform penetration before surgery.

Mediastinal UCD is treated with surgical resection, which includes open surgery and video-assisted thoracoscopy. A previous study has shown video-assisted thoracoscopy to be not useful (7). However, there have been cases of effective thoracoscopic resection in recent years (8, 9). Video-assisted thoracoscopy is a good option for UCD that is located in the anterior mediastinum or upper mediastinum and has distinct boundaries and does not oppress the heart or blood vessels. The tumor in our case was near the pulmonary arteries and left atrium with no clear boundaries. Therefore, VATS was not used in our case; we believed that median sternotomy was safer for the patient.

Surgical resection of UCD is complicated by excessive blood loss because CD is highly vascularized. Preoperative embolization can be used as an adjunct to avoid intraoperative bleeding (10). In our case, we used CPB to guarantee complete resection of the tumor and the safety of the patient. The application of CPB is of great contribution to intraoperative dynamic stability and meticulous surgical bleeding control. Moreover, to reduce the chances of rupture during operation, the pressure on the pulmonary and cardiac arteries can be relieved by CPB. In our case, the patient did not require any blood transfusion. Thus, our experience supports the application of CPB as a safe and effective technique for the successful surgical treatment of UCD.

## Conclusion

CD is a rather uncommon condition. Pathological diagnosis is the gold standard, as radiographic examination is not specific. For UCD, surgical resection is the treatment of choice. The application of CPB is a useful and effective technique for successful surgical treatment.

## Data Availability

The original contributions presented in the study are included in the article, further inquiries can be directed to the corresponding author/s.
